# Retraction Note to: Improving the measurement of alexithymia in autistic adults: a psychometric investigation and refinement of the twenty-item Toronto Alexithymia Scale

**DOI:** 10.1186/s13229-021-00446-6

**Published:** 2021-05-30

**Authors:** Zachary J. Williams, Katherine O. Gotham

**Affiliations:** 1 Medical Scientist Training Program, Vanderbilt University School of Medicine, Nashville, TN USA; 2 Department of Hearing and Speech Sciences, Vanderbilt University Medical Center, 1215 21st Avenue South, Medical Center East,Room 8310, Nashville, TN 37232 USA; 3 Vanderbilt Brain Institute, Vanderbilt University, Nashville, TN USA; 4 Frist Center for Autism and Innovation, Vanderbilt University, Nashville, TN USA; 5 Department of Psychology, Rowan University, Glassboro, NJ USA

## Retraction Note to: Molecular Autism (2021) 12:20 10.1186/s13229-021-00427-9

The authors have retracted this article because they did not have permission from the copyright holders to use and adapt the twenty-item Toronto Alexithymia Scale (TAS-20). This retraction is not related to the results or conclusions presented in the article, which remain scientifically valid. The authors are preparing a revised manuscript in accordance with requests from the TAS-20 copyright holders, and they plan to resubmit this revised version to this Journal for peer review. Both authors agree with this retraction.

